# Overcoming diagnostic challenges in desmoplastic fibroma of the scapula: a rare case report

**DOI:** 10.1093/omcr/omad057

**Published:** 2023-08-20

**Authors:** Mehran Razavipour, Hadi Akhlaghi, Amirsaleh Abdollahi

**Affiliations:** Orthopedic Research Center, Mazandaran University of Medical Sciences, Sari, Iran; Orthopedic Research Center, Mazandaran University of Medical Sciences, Sari, Iran; Mazandaran University of Medical Sciences, Sari, Iran

## Abstract

Desmoplastic fibroma (DF) is an aggressive benign tumor that commonly affects long tubular bones. Also, the skull, mandible, pelvis and spine involvement have been reported. However, its occurrence in the scapula is extremely rare. In this case report, we present the challenging diagnosis and successful treatment of DF in a 27-year-old woman who had been experiencing worsening pain in her right shoulder for 5 years. Plain radiographs and magnetic resonance imaging revealed a lucent, trabeculated and expansile infiltrative lesion, disrupting the posterior cortex and extended to the posterior soft tissue. After ruling out malignancy through a core needle biopsy, the patient underwent wide surgical resection of the tumor, which involved a hemi-scapulectomy. And histologic diagnosis consistent with DF, no postoperative radiation was administered. Remarkably, the patient became pain-free just 2 weeks after surgery. Follow-up examinations, X-rays and computed tomography scans conducted 6 weeks, 6 months and 18 months after surgery revealed no signs of recurrence.

## INTRODUCTION

Desmoplastic fibroma (DF) is one of the primary benign-aggressive bone tumors, which includes 0.06% of bone tumors and 0.3% of all benign bone tumors [1, 2]. Since 1958, almost 200 cases of DF of the bone have been reported [1]. It is a very rare lytic tumor with local invasion and non-metastatic nature, which has the highest incidence in the second and third decades of life and occurs equally in men and women [1, 3]. Pathologically, DF is very similar to the desmoid tumor of soft tissue and contains many wavy fibroblasts that float in the collagen matrix [4]. The most common sites of involvement are the mandible, femur, pelvis, radius and tibia [1, 2]. The most common symptoms include pain and swelling; in 12% of patients, pathologic fracture is the First Clinical Manifestation of disease, and in asymptomatic cases, the tumor is discovered accidentally [5].

This study aimed to discuss the clinical, histological, immunohistochemical and radiographic characteristics of this rare neoplasm; a few case reports about DF have been published.

## CASE REPORT

A 27-year-old woman presented to the Orthopedic clinic at the Imam Khomeini Hospital, Sari, Iran, in December 2020 with a 5-year history of pain in the right shoulder without follow-up. The pain had initially been dull, activity-dependent and relieved by the occasional oral administration of an analgesic. The pain gradually progressed in intensity during the 6 months before the visit. The patient reported continuous consumption of analgesics. The patient was a housekeeper who had not engaged in any strenuous or specific physical activity. There was no family history of lesions or masses. During examination, there is no redness, warmth or nodules present upon palpation of the skin. There is no evidence of pigmentation.

### Physical examination

No deformity was found in the right shoulder; in palpation, a tender 2 × 2 cm mass was felt in the scapula area. The range of Motion was painful in all directions, but there was no limit to the passive Range of Motion. The neurologic exam was normal, and no hypoesthesia was detected.

### Imaging data

**Figure 1:**
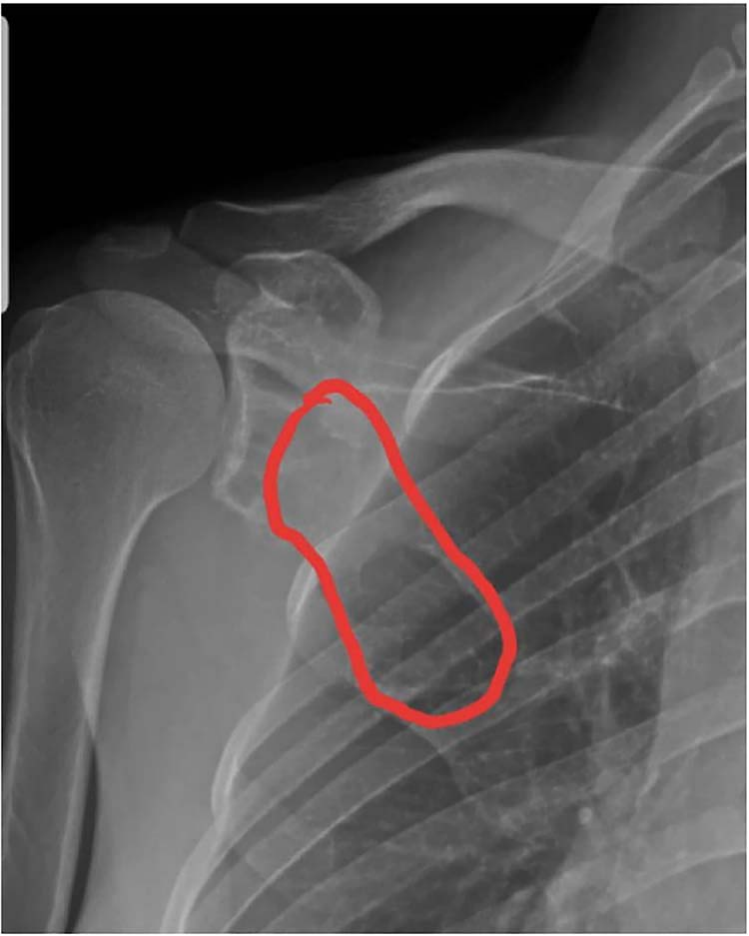
X-ray of the right shoulder showing an ill-defined, eccentric, lucent lesion in the right scapula.

**Figure 2:**
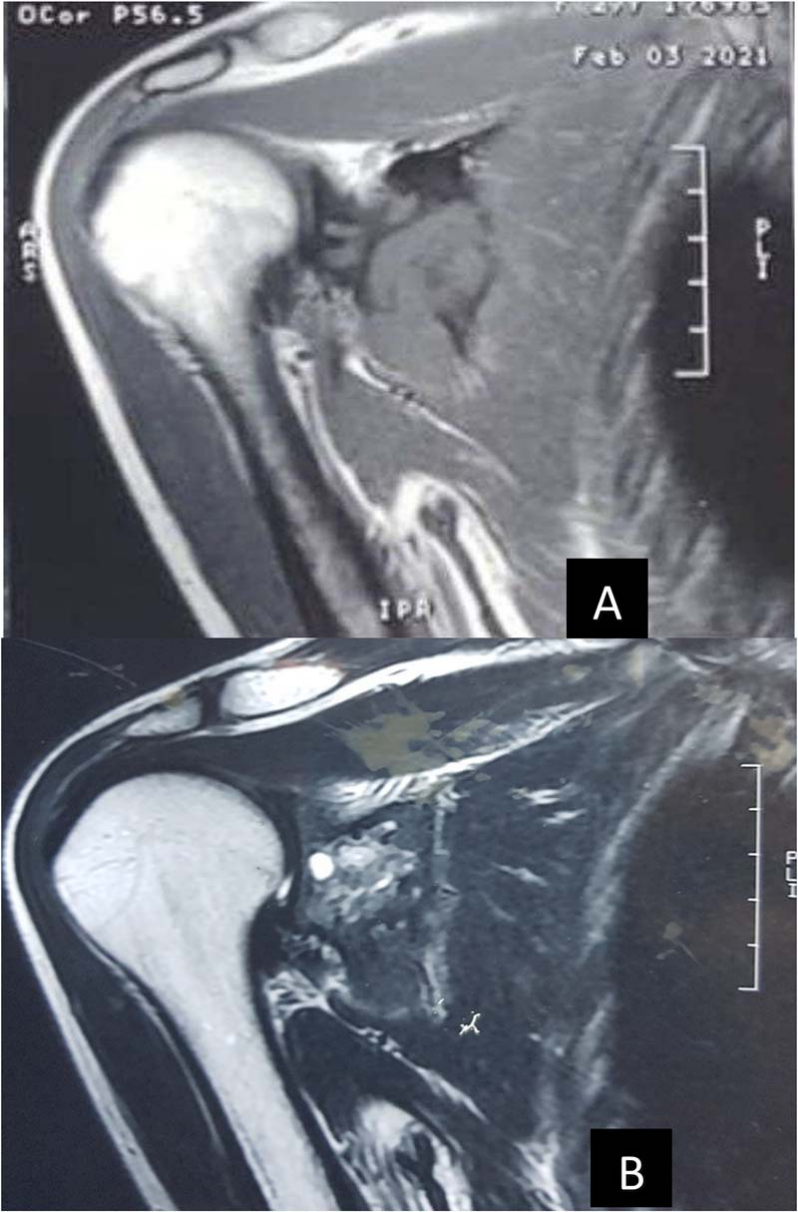
MRI images of the right shoulder displayed a high signal intensity lesion, (**A**) T1-weighted MRI demonstrates decreased signal at the right scapula and (**B**) T2-weighted MRI.

**Figure 3:**
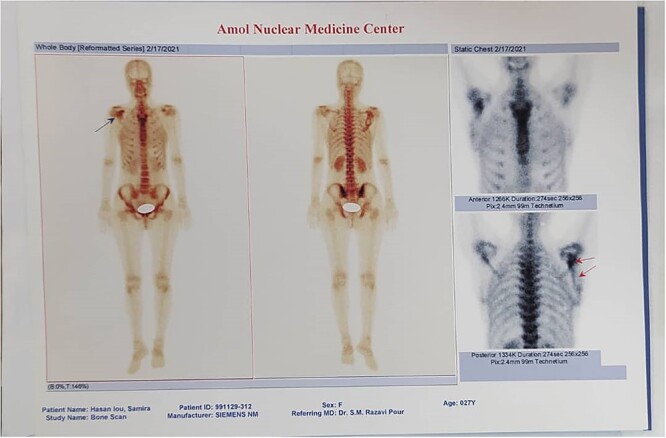
Whole-body bone scan revealed irregular tracer activity in the lateral border of right scapula.

Plain radiographs revealed a trabeculated, lucent, expansile lesion with cortical destruction in the scapula, and a soft-tissue mass also was seen (Fig. 1). Computed tomography (CT) scan confirmed expansile, lytic in the body of the right scapula that extended to the coracoid. Right shoulder magnetic resonance imaging (MRI) showed an expansile infiltrative lesion involving the scapula with disruption of the posterior cortex and extension to the posterior soft tissue. The coracoid process was also involved (Fig. 2). Whole-body bone scintigraphy reported irregular tracer activity in the lateral border of the right scapula, which could be a primary bone lesion and less likely a malignant tumor (Fig. 3). Lab data were normal (Ca = 10.2, *P* = 3, Alkp = 117, PTH = 30.5, ESR = 5, CRP = 3.3).

### Biopsy

An expert intervention radiologist performed a CT-guided core needle biopsy, and no evidence of malignancy was seen, and the section demonstrated portions of tissue composed of degenerated bone.

### Surgical data

**Figure 4:**
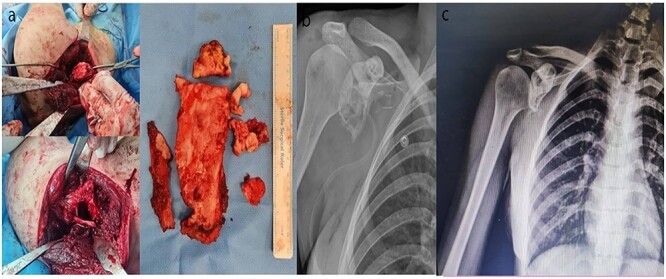
(**a**) Wide resection of the tumor through Judet approach; (**b**) post-op X-ray after 6 weeks and (**c**) post-op X-ray after 18 months.

Wide surgical resection of the tumor (hemi-scapulectomy) was performed with the patient under general anesthesia. Through Judet Approach (Fig. 4). The removed tumor was sent for further histological examination to the pathology department.

**Figure 5:**
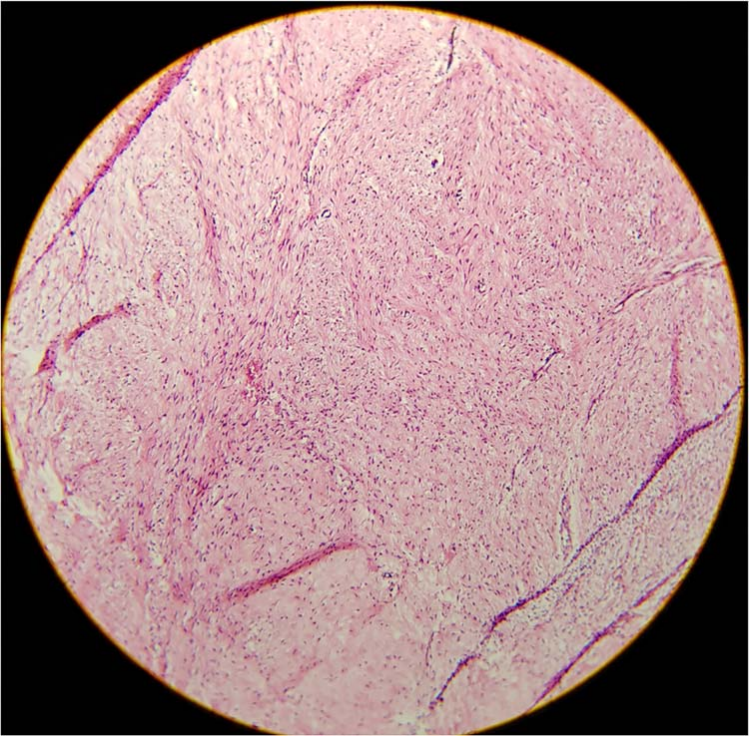
Histology of biopsy showed mesenchymal neoplasm composed of whorled bundles of well-differentiated spindle-shaped cells with rare mitotic activity and foci of myxoid stromal changes (hematoxylin and eosin; original magnification 40x).

Histological examination of the bone specimen following the tumor resection revealed mesenchymal neoplasm composed of whorled bundles of well-differentiated spindle-shaped cells with rare mitotic activity and foci of myxoid stromal changes and some bony trabeculae also noted; the appearance was in accordance with a fibroblastic desmoid tumor of the bone a (desmoid type fibromatosis) (Fig. 5). The immunohistochemical results revealed Actin (+), SMA (partially +), CD34 (+ in the vessel wall), EMA (−), Desmin (+), Ki-67 (+1%), S-100 (−) and Vimentin (+), which resulted in a diagnosis of DF of bone.

No postoperative radiation of the lesion was performed. The patient became pain-free after 2 weeks. Postoperative follow-up examinations and X-rays, and CT-scan 6 weeks and 6 and 18 months after surgery showed no signs of recurrence.

## DISCUSSION

DF of bone is an extremely rare, locally aggressive benign bone tumor. The WHO described the microscopic appearance of DF as being composed of slender, spindle-to-stellate cells with minimal cytological atypia, few mitoses and an abundant collagenous matrix [5, 6], so this infiltrative pattern causes DF to be considered the bony counterpart of the desmoid tumor of soft tissue. However, DF can genetically be distinguished from desmoid-type fibromatosis [6].

DF has been reported in all age groups but is more common in the second and third decades [2] as our patient.

The long tubular bones are often involved, but the involvement of the skull, mandible, pelvis and spine has been reported [1–3, 7]. Very few studies have been reported about DF of scapula. Taketo *et al*. [8] reported a 65-year-old woman who had DF of right scapula that was incidentally identified by fluorodeoxyglucose-positron emission tomography. However, in our case, constant worsening pain of our patient with imaging and biopsy led us to diagnose this disease. Pain is typically the chief complaint in all reported cases [7, 9] as our case.

On plain radiography, the tumor appears to be a well-circumscribed lytic lobulated lesion with a narrow transition zone and, frequently, a thin rim of reactive bone with or without cortical destruction [7, 10]. On MRI, DF, similar to other fibrous tumors, shows low signal intensity on T1- and T2-weighted images [10]. The differential diagnosis includes benign lesions, such as fibrous dysplasia, simple bone cyst, aneurysmal bone cyst, non-ossifying fibroma, eosinophilic granuloma and chondro-myxofibroma. However, if cortical destruction and a soft tissue mass are noted, as in our case, malignant tumors such as fibrosarcoma, intra-osseous osteosarcoma and metastases must be considered [6, 7]. Open biopsy is the gold standard, with 98% accuracy for diagnosis of the majority of musculoskeletal tumors, including DF; an image-guided percutaneous biopsy is becoming an increasingly accepted method of choice for initial biopsy with accuracy estimated between 78 and 98.4%. Levrini *et al*. [1] reported a 36-year-old female patient whose DF was diagnosed in her based on a non-invasive method by CT-guided core needle biopsy.

Metastasis and malignant transformation have not been reported in patients with DF, but local recurrence is common after simple curettage, between 37% and 72% [6, 10], so wide resection is the preferred treatment method. Especially in the presence of extra-osseous tumor growth, which is a poor prognostic sign [6]. However, aggressive extended curettage may be a reasonable option in selected patients to preserve better function [6, 7].

## CONCLUSION

Through this case report, we highlight the importance of early detection, careful evaluation and multidisciplinary management of DF, particularly when it occurs in rare sites such as the scapula.

## Data Availability

The data underlying this article are available in the article.
